# Antioxidant, Anti-Inflammatory, and Microbial-Modulating Activities of Nutraceuticals and Functional Foods 2018

**DOI:** 10.1155/2018/3824509

**Published:** 2018-07-12

**Authors:** Ilaria Peluso, Débora Villaño Valencia, C.-Y. Oliver Chen, Maura Palmery

**Affiliations:** ^1^Research Centre for Food and Nutrition, Council for Agricultural Research and Economics (CREA-AN), Rome, Italy; ^2^Universidad Católica San Antonio de Murcia (UCAM), Murcia, Spain; ^3^Antioxidants Research Laboratory, Jean Mayer USDA Human Nutrition Center on Aging, Tufts University, Boston, MA, USA; ^4^Department of Physiology and Pharmacology, “V. Erspamer”, La Sapienza University of Rome, Rome, Italy

The antioxidant and anti-inflammatory activities of nutraceuticals are current research topics in the area of the management and prevention of oxidative stress-related diseases. This issue includes preclinical research aimed to evaluate the effect of pure compounds (P. Aguilar-Alonso et al., “Evaluation of Oxidative Stress in Cardiomyocytes during the Aging Process in Rats Treated with Resveratrol”; W.-R. Hao et al., “Cafestol Inhibits Cyclic-Strain-Induced Interleukin-8, Intercellular Adhesion Molecule-1, and Monocyte Chemoattractant Protein-1 Production in Vascular Endothelial Cells”; and H. Sun et al., “The In Vitro Antioxidant Activity and Inhibition of Intracellular Reactive Oxygen Species of Sweet Potato Leaf Polyphenols”), extracts from plant (W. Huang et al., “Antioxidant and Anti-Inflammatory Effects of Blueberry Anthocyanins on High Glucose-Induced Human Retinal Capillary Endothelial Cells”; Z. Liao et al., “Protective Role of Antioxidant Huskless Barley Extracts on TNF-*α*-Induced Endothelial Dysfunction in Human Vascular Endothelial Cells”; H. Sun et al. “The In Vitro Antioxidant Activity and Inhibition of Intracellular Reactive Oxygen Species of Sweet Potato Leaf Polyphenols”; and K. Wei Chan et al., “Defatted Kenaf (*Hibiscus cannabinus* L.) Seed Meal and Its Phenolic-Saponin-Rich Extract Protect Hypercholesterolemic Rats against Oxidative Stress and Systemic Inflammation via Transcriptional Modulation of Hepatic Antioxidant Genes”), fungal (X. Wang et al., “Antifatigue Potential Activity of *Sarcodon imbricatus* in Acute Excise-Treated and Chronic Fatigue Syndrome in Mice via Regulation of Nrf2-Mediated Oxidative Stress) or animal (Y. Zhang et al., “Acute Toxicity, Antioxidant, and Antifatigue Activities of Protein-Rich Extract from *Oviductus ranae*”) sources, and a defatted kenaf seed meal (DKSM) (K. Wei Chan et al.).

*In vitro* data illustrate that blueberry anthocyanin-rich extract (W. Huang et al.), huskless barley extract (Z. Liao et al.), and sweet potato leaf polyphenols (H. Sun et al.) improve the redox status by reducing intracellular reactive oxygen species (H. Sun et al. and W. Huang et al.) and/or by increasing the capacity of antioxidant enzymes (W. Huang et al. and Z. Liao et al.). Two of these research papers (Z. Liao et al. and W. Huang et al.) also show that constituents in the huskless barley extract and sweet potato leaf polyphenols enable the reduction of monocyte chemotactic protein 1 (MCP-1), vascular cell adhesion molecule 1 (VCAM-1), intercellular adhesion molecule-1 (ICAM-1), and/or nuclear factor-kappa B (NF-*κ*B) in human endothelial cells, which are in line with the concerted modulation of nuclear factor-erythroid 2-related factor 2 (Nrf2) and NF-*κ*B in inflammation and oxidative stress. However, H. Sun et al. note that the inhibitory effect of individual polyphenolic compounds present in sweet potato leaves on the intracellular ROS is not related to their antioxidant activity, suggesting that the polyphenolic compounds may attenuate the sources of ROS generation. However, the precaution must be taken in the extrapolation of the *in vitro* findings to humans because most absorbed polyphenols are present in metabolized forms. Moreover, the protein-rich extract of *Oviductus ranae* (PEOR) displays a strong antioxidant effect in ethanol-induced oxidative stress mice model, despite its weak radical scavenging and ferric-reducing capacities *in vitro* (Y. Zhang et al.).

Both PEOR (Y. Zhang et al.) and *Sarcodon imbricatus* (X. Wang et al.) have an antifatigue effect in mice and upregulate antioxidant enzymes. In particular, *Sarcodon imbricatus* (X. Wang et al.) induces Nrf2, superoxide dismutase (SOD), haem oxygenase-1 (HO-1), and catalase (CAT). Upregulation of HO-1 has been reported in vascular endothelial cells treated with the diterpene cafestol (W.-R. Hao et al.). The latter, found in coffee, can inhibit ICAM-1, MCP-1, and mitogen-activated protein kinases (MAPK) pathway. Antioxidant and anti-inflammatory properties of DKSM and its phenolic-saponin-rich extract (PSRE) observed in hypercholesterolemic rats are likely modulated via the activation of the Nrf2-antioxidant responsive element (ARE) pathway (K. Wei Chan et al.). On the contrary, in rats treated with resveratrol, the decrease in nitric oxide and lipoperoxidation in the cardiac tissues is not accompanied by the induction of antioxidant enzymes (CAT and SOD).

In addition to the antioxidant and anti-inflammatory effects of huskless barley extracts, the polysaccharide extracts inhibit the synthesis of angiotensin-converting enzyme (ACE) *in vitro* (Z. Liao et al.), with the alkaline extracts being more pronounced probably due to the abundance of phenolic compounds as compared to the water extracts. The modulation of ACE expression is also found with blueberry anthocyanins and anthocyanidins (W. Huang et al.), but the effect is divergent, for example, malvidin downregulates the expression and some malvidin glycosides result in the upregulation.

Therefore, caution must be taken when interpreting preclinical data that evaluate the effects of natural functional compounds on antioxidation, anti-inflammation, and other pharmacological actions. In this context, E. Toti et al. (Non-Provitamin A and Provitamin A Carotenoids as Immunomodulators: Recommended Dietary Allowance, Therapeutic Index, or Personalized Nutrition?) underscore that preclinical evidence must be confirmed in human interventions before any recommendations can be made for carotenoids. This point can also be extended to polyphenols and other dietary agents. For example, both *β*-carotene and lycopene at pharmacological doses affect immune functions. However, large clinical trials do not support *β*-carotene supplementation. On the other hand, although lycopene supplementation for regulation of immunity seems more promising than *β*-carotene, more robust human studies with adequate power and duration are needed in order to confirm this effect.

In conclusion, we hope that this special issue adds knowledge of preclinical data of the potential health effects of nutraceuticals. However, these results only provide supports for future studies, particularly human trials, but not give indications for supplementation.

